# Letter to the Editor, Reacting to: “*APOE* ε4 Carriers Have a Greater Propensity to Glycation and sRAGE Which Is Further Influenced by RAGE G82S Polymorphism”

**DOI:** 10.1093/gerona/glaa037

**Published:** 2020-02-03

**Authors:** Sanne S Mooldijk, Jinluan Chen, M Arfan Ikram, M Carola Zillikens

**Affiliations:** 1 Department of Epidemiology, Erasmus MC, University Medical Center Rotterdam, The Netherlands; 2 Department of Internal Medicine, Erasmus MC, University Medical Center Rotterdam, The Netherlands

To the editor,

In their article recently published in this Journal, Deo and colleagues suggest that *APOE ε4* carriers have greater propensity to glycation than noncarriers, which may play a role in the pathophysiology of dementia (1).

They base their conclusions on findings that *APOE ε4* carriers had higher concentrations of serum advanced glycation end products (AGEs), which are considered harmful, and of soluble receptor for *AGEs* (*sRAGE*), considered to be protective, than noncarriers. The authors interpret higher *sRAGE* to be a defensive mechanism in response to increasing *AGEs*. Furthermore, they found lower *sRAGE* levels in individuals with the Gly82Ser variant in the *RAGE* gene.

Intrigued by their work, we set out to replicate and further expand their findings in nondemented and nondiabetic subsamples of the population-based Rotterdam Study (2) with appropriate measurements. Similar to Deo and colleagues, we measured sRAGE and carboxymethyllysine (CML) and additionally EN-RAGE, which is a RAGE ligand, in blood samples from 894 participants obtained between 1997 and 1999. Moreover, using skin auto fluorescence (SAF), we obtained a marker of AGE accumulation in the skin (3–5) in 2,439 persons between 2012 and 2016. On average, our population was 72.6 years old (*SD* 8.7) and 56.7% was female.

**Figure 1.**
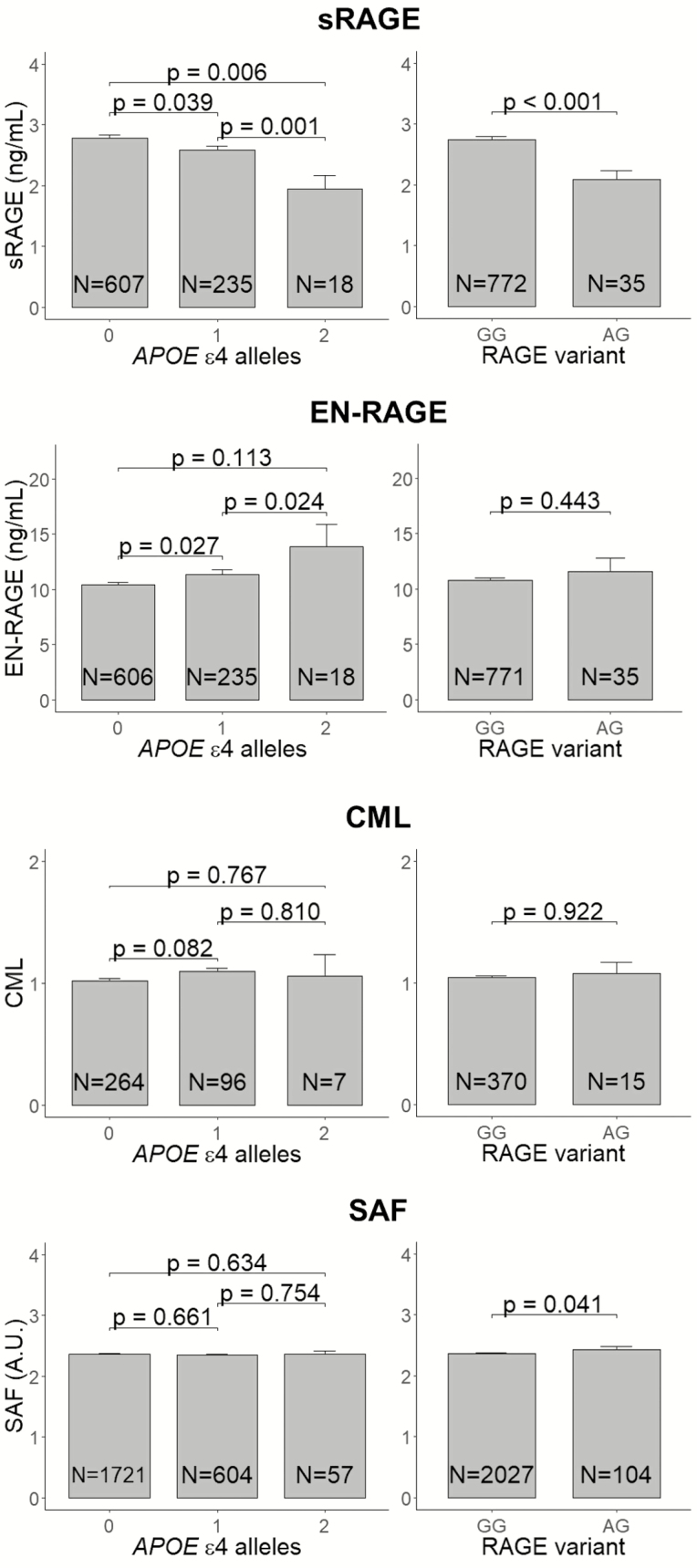
sRAGE, EN-RAGE, CML, and SAF levels by APOE ε4 carrier status and by RAGE variant status; among nondemented, nondiabetics mean levels of sRAGE*, EN-RAGE*, CML, and SAF by APOE ε4 carrier status (left panels) and by RAGE variant carrier status (right panels). Error bars represent the standard error of means. CML was quantified by the raw area-under-the-curve of its peak and the median was set to 1.0, which preserved variation among samples. RAGE gene status is reflected by presence of the Gly82Ser (G82S) polymorphism (A reflects the variant). *p* Values are obtained using ANCOVA test with adjustment for age, sex, and BMI. *For non-normally distributed variables (sRAGE and EN-RAGE), values were log-transformed before calculation of means, standard errors and *p* values. Means and standard errors as presented in the plots were back transformed by taking the exponent of these values. ANCOVA = analysis of covariance; BMI = body mass index; CML = carboxymethyllysine; SAF = skin auto fluorescence.

APOE genotype was determined using polymerase chain reaction on coded DNA or with a bi-allelic TaqMan assay. Presence of the Gly82Ser variants in the RAGE gene was obtained from the SNPs array data imputed to the reference dataset (Haplotype Reference Consortium [HRC] r1.1) (2). Figure 1 shows the levels of the biomarkers by *APOΕ ε4* carrier status. sRAGE was lower and EN-RAGE was higher among *APOΕ ε4* carriers than noncarriers. We found slightly higher CML levels for carriers of one *APOE ε4* allele compared to noncarriers. SAF did not differ by *APOE ε4* carrier status. Among carriers of the RAGE G82S variant, the level of sRAGE was lower and the levels of EN-RAGE and SAF were higher (Figure 1).

In line with Deo and colleagues, we found that *APOE* ε4 carriers and noncarriers have different AGE and RAGE profiles. Carriers had higher levels of EN-RAGE and CML than noncarriers, suggesting a harmful effect of *APOE* ε4 on the AGE-RAGE profile. However, compared to the Deo and colleagues’ study, we found a contrasting pattern for sRAGE, namely lower levels among carriers than noncarriers. This discrepancy may be explained by differences in characteristics of the study populations. For instance, the study population of Deo and colleagues was younger and consisted of healthy volunteers, which may influence the AGE-RAGE profile and the impact of *APOE ε4*. Moreover, sRAGE can be formed in two different ways, firstly via cleavage from the original receptor (RAGE) and secondly via direct transcription of sRAGE (6). Both products act as a decoy for RAGE ligands, although they may have different causes for an increase in level. Among older adults, with more comorbidities and with normal aging, there may be more proinflammatory RAGE ligands, leading to high usage of sRAGE as a decoy. This may especially be the case among *APOE ε4* carriers, due to increased inflammation (7,8). With regard to the RAGE G82S variant, our results were similar to what Deo and colleagues reported.

Concluding, we found a more harmful profile of AGE and RAGE markers for *APOE* ε4 carriers than for noncarriers. Based on our results, we could not confirm the suggested idea of an increase in sRAGE as a defensive response. As Deo and colleagues also proposed, APOE and the AGE-RAGE system may have a (combined) role in dementia pathophysiology, which may be addressed by future studies. Future studies may also distinguish between the two types of sRAGE and investigate change in levels over time to gain a more in depth knowledge about the role of sRAGE.
